# The CBL–CIPK Pathway in Plant Response to Stress Signals

**DOI:** 10.3390/ijms21165668

**Published:** 2020-08-07

**Authors:** Xiao Ma, Quan-Hui Li, Ya-Nan Yu, Yi-Ming Qiao, Saeed ul Haq, Zhen-Hui Gong

**Affiliations:** 1 College of Horticulture, Northwest A&F University, Yangling 712100, China; mxiao26@163.com (X.M.); liquanhui_2008@163.com (Q.-H.L.); 2018050315@nwafu.edu.cn (Y.-N.Y.); qym941226@163.com (Y.-M.Q.); Saeed_ulhaq@nwsuaf.edu.cn (S.u.H.); 2 Academy of Agricultural and Forestry Sciences, Qinghai University, Xining 810016, China

**Keywords:** abiotic stress, biotic stress, CBL, CIPK, signaling

## Abstract

Plants need to cope with multitudes of stimuli throughout their lifecycles in their complex environments. Calcium acts as a ubiquitous secondary messenger in response to numerous stresses and developmental processes in plants. The major Ca^2+^ sensors, calcineurin B-like proteins (CBLs), interact with CBL-interacting protein kinases (CIPKs) to form a CBL–CIPK signaling network, which functions as a key component in the regulation of multiple stimuli or signals in plants. In this review, we describe the conserved structure of CBLs and CIPKs, characterize the features of classification and localization, draw conclusions about the currently known mechanisms, with a focus on novel findings in response to multiple stresses, and summarize the physiological functions of the CBL–CIPK network. Moreover, based on the gradually clarified mechanisms of the CBL–CIPK complex, we discuss the present limitations and potential prospects for future research. These aspects may provide a deeper understanding and functional characterization of the CBL–CIPK pathway and other signaling pathways under different stresses, which could promote crop yield improvement via biotechnological intervention.

## 1. Introduction

As sessile organisms, plants endure varied climate conditions incessantly and cannot escape their local environment as animals can. Hence, plants have evolved complex mechanisms that enable them to survive environmental stresses [1]. Environmental stresses caused by climate conditions can be broadly divided into abiotic and biotic stresses, such as salt stress, water deficiency, extreme temperatures (i.e., cold and heat), pathogens, and nematodes, each of which results in declined crop productivity worldwide [2].

Calcium functions as a ubiquitous second messenger and a regulator in most signaling networks that responds to plant hormones and various stresses. In response to complex stimuli, the concentration of Ca^2+^ in the cytoplasm can increase from 10^−7^ M to 10^−6^ M [3]. The membranes and organelles are damaged when the concentration reaches 10^−4^ M [4]. To regulate Ca^2+^ homeostasis in the cytosol, various relative channels and transporters have been gradually identified. Emerging channels and transporters such as Ca^2+^-ATPases (ACAs), cation/proton exchangers (CAXs), cation/Ca^2+^ exchangers (CCXs), glutamate-like receptors (GLRs), cyclic nucleotide-gated channels (CNGCs), and vacuolar two-pore channels (TPCs), play substantial roles in regulating the influx and efflux of Ca^2+^ [5,6,7,8,9,10]. Further, intracellular organelles and subcompartments also contribute to Ca^2+^ homeostasis [11,12]. All these stimuli-specific fluctuations of distinct changes in cellular Ca^2+^ concentration spatially and temporally are referred to as calcium signatures [2,3,13]. Meanwhile, the elevation of Ca^2+^ concentration in the cytoplasm and the accumulation of reactive oxygen species (ROS) regulate each other in concert under stress conditions, suggesting a vital connection between Ca^2+^ signaling and ROS signaling [14].

**Figure 1:**
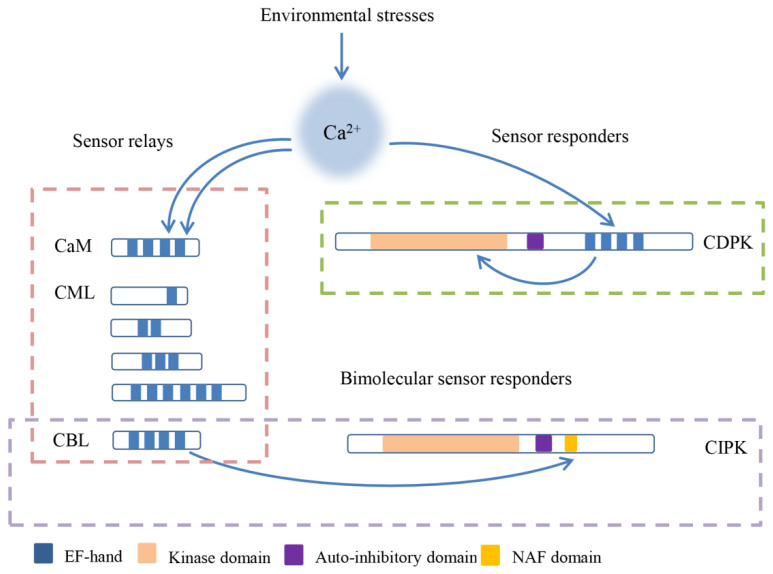
The schematic diagram and classification of calcium sensors in plants. Calcium sensors combine with the Ca^2+^ by EF-hand, and then activate the binding proteins or themselves to regulate the downstream. calmodulins (CaMs), CaM-like proteins (CMLs), and calcineurin B-like proteins (CBLs) are sensor relays that interact with Ca^2+^-dependent protein, and calcium-dependent protein kinases (CDPKs) are sensor responders which contain the protein kinase domain. The special sensors CBLs interact with CBL-interacting protein kinases (CIPKs) and form the bimolecular sensor responder.

The initial decoding of calcium signals is captured by calcium sensors, which contain a helix-loop-helix domain called an EF-hand (Elongation Factor-hand) motif and consist of sensor relays and sensor responders [15,16]. Various calcium binding proteins such as calmodulins (CaMs), CaM-like proteins (CMLs), calcineurin B-like proteins (CBLs), and calcium-dependent protein kinases (CDPKs) form calcium sensors in plants (Figure 1) [2]. CDPKs combine with EF-hand motifs and a protein kinase to sense Ca^2+^ and respond to Ca^2+^, respectively, and they can be categorized as sensor responders [15,17,18,19]. CaMs, CMLs, and CBLs are relatively small proteins without enzymatic function, and they play roles via Ca^2+^-dependent protein–protein interactions as parts of sensor relays [20,21]. In contrast with other calcium sensors, CMLs harbor a number of unfixed EF-hand motifs [21,22].

In order to understand CBLs, calcineurin, the Ser/Thr phosphatase that is activated by Ca^2+^ and CaM, is important to consider [23]. In animals and fungi, the regulatory subunit calcineurin B (CnB) and CaM bind with Ca^2+^ and interact with the catalytic subunit calcineurin A (CnA) [24,25]. CBLs are similar to CnB but only exist in plants. With a structure similar to that of CnB-CnA, CBLs specifically combine with CBL-interacting protein kinases (CIPKs) to form bimolecular sensor responders involved in Ca^2+^ signaling [1,16,25]. Notably, the CnB-CnA and CBL–CIPK complexes occur in animals and plants, and understanding their respective functions merits further research.

CBL–CIPK signaling has been extensively studied since the original identification of CBLs and CIPKs in *Arabidopsis thaliana* [23,26]. Recent studies have found that CBL–CIPK signaling takes part via the membrane system and the transport of ions such as sodium, potassium, magnesium, and nitrate [13,27,28,29,30]. The CBL–CIPK pathway is also involved in abscisic acid (ABA) signaling and alters stomatal movement [31]. In addition, the CBL–CIPK network participates in multiple biotic and abiotic stresses, including drought, heat, cold, *Phytophthora capsici*, and stripe rust fungus [32,33,34,35]. Here, we present the typical structure and mechanisms of CBLs and CIPKs and summarize the current progress in understanding the functions of the CBL–CIPK signaling system in order to provide a framework for future functional dissection.

## 2. Structure and Classification of CBLs and CIPKs

**Figure 2:**
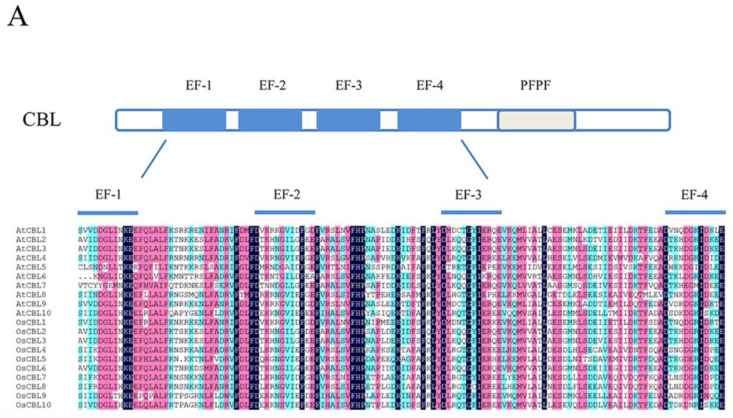

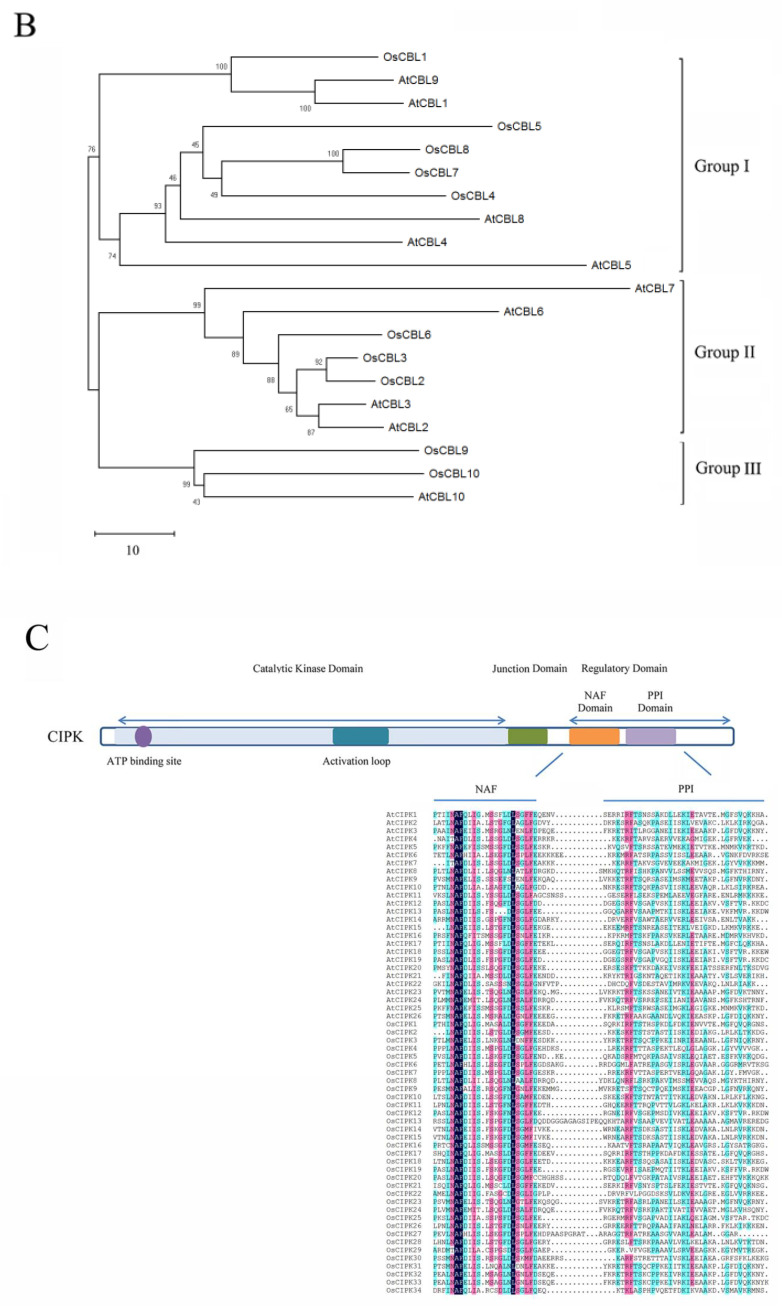

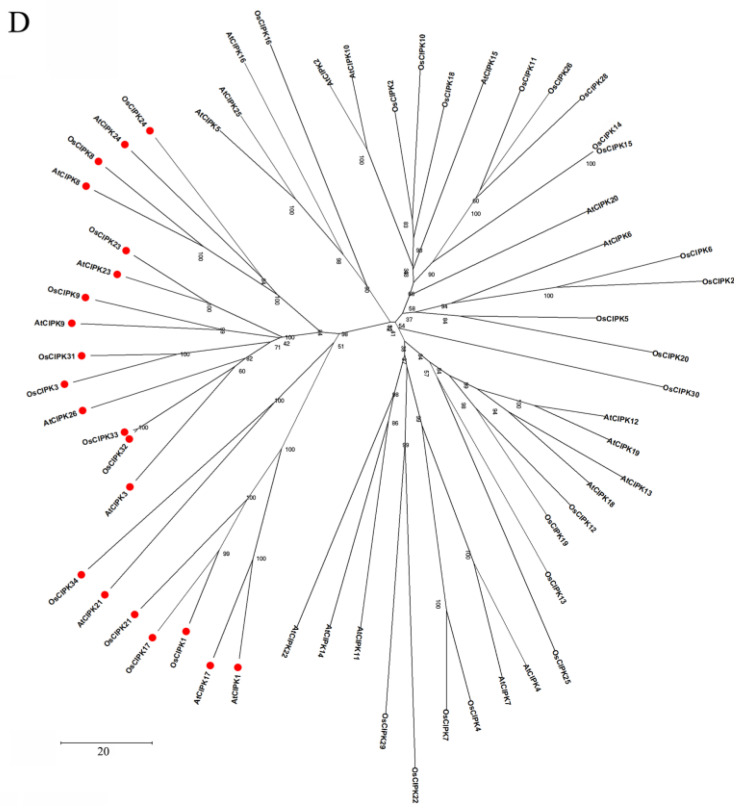
The domain structure and phylogenetic relationships of CBLs and CIPKs in *Arabidopsis* and rice (*Oryza sativa*). (**A**) CBLs contain four EF-hands that are separated by short amino acids with conserved numbers; (**C**) CIPKs contain conserved NAF motif and protein phosphatase interaction (PPI) motif which interact with CBLs and PP2Cs, respectively; (**B**,**D**) The phylogenetic relationships combined the classification of CBLs and CIPKs. The intron-rich CIPKs are distinguished with a red dot.

CBLs and CIPKs were first studied in the model plant *Arabidopsis*, and these families have since then also been identified in many species, such as *Oryza sativa*, *Triticum aestivum*, *Populus trichocarpa*, *Capsicum annuum*, *Brassica napus*, *Manihot esculenta*, *Camellia sinensis*, *Physcomitrella patens, Selaginella moellendorffii,* and *Vitis vinifera* [13,29,34,36,37,38,39,40,41,42,43,44] (Table 1). Clarification of the structural features of CBLs and CIPKs has contributed to the understanding of the mechanisms of the signaling system (Figure 2).

Like other calcium sensors, CBLs are similar in size and contain four Ca^2+^-binding EF-hand motifs separated by short amino acids that are conserved in number [2,19] (Figure 2A). The sequence of the conserved EF-hand motif consists of 12 residues conforming to the model X•Y•Z•-Y•-X••-Z. In the model, the letters and the dots represent the metal coordinated ligands and the intervening residues, respectively [45,46]. Commonly, the amino acids in canonical EF-hand motifs are Asp, Asp, Asp, Thr, Asp, and Glu in the position of coordinated ligands [46]. Although there are four EF-hand motifs in CBLs, not all of them have the canonical sequence for Ca^2+^ binding [1]. Specifically, AtCBL1 and AtCBL9 contain two canonical EF-hand motifs, while AtCBL6, AtCBL7, and AtCBL10 have one canonical EF-hand motif, and others have none [39]. Some data show that the calcium-dissociation constants of CBLs are generally lower than those of other sensor proteins, which is caused by the absence of canonical EF-hand domains [47,48]. Furthermore, CBLs possess a PFPF motif harboring a conserved serine residue, which is phosphorylated by CIPK at the C-terminus [2,49]. Furthermore, in algal CBLs, some PFPF motifs contain Asp and Glu instead of Ser [25]. Remarkably, some CBLs harbor the *N*-myristoylation and palmitoylation sites that usually bind cell membranes [39,50].

CBLs can be classified into three groups depending on the structure of the N-terminus [51]. The conserved structure of CBLs can influence their specific localizations and functions. CBLs in the first group harbor a relatively short N-terminal domain with a dual-lipid modification motif, such as AtCBL1, AtCBL4, AtCBL5, OsCBL5, OsCBL7, and OsCBL8 in *Arabidopsis* and rice, respectively. The second group possesses a tonoplast targeting sequence (TTS) in the N-terminal domain, comprising AtCBL2, AtCBL3, AtCBL6, AtCBL7, OsCBL2, OsCBL3, and OsCBL6 [51,52]. Compared with other CBLs, AtCBL10, OsCBL9, and OsCBL10 harbor a transmembrane helix that belongs to the third group [35,53,54]. Meanwhile, the grouping accords with currently understood phylogenetic relationships (Figure 2B).

CIPKs are Ser/Thr protein kinases that are targeted by CBLs to participate in calcium signaling, and they can also be classified as SNF1-related kinases 3 (Sucrose non-fermenting-1-related kinases, group 3; SnRK3) according to their evolutionary relationships [26,55,56]. CIPKs possess an N-terminal catalytic kinase domain with an ATP binding site and an activation loop (Figure 2C). Following the junction domain, the regulatory domain contains a unique NAF/FISL motif and protein phosphatase interaction (PPI) motif at its C-terminus [57]. Phosphorylation at conserved residues of the activation loop may be critical to regulating enzyme activity [58]. While the conserved residues Ser^156^, Thr^168^, and Tyr^175^ are changed to Asp in the activation loop, AtCIPK24 could be activated [59,60]. Besides the activation loop, Ser^228^ is autophosphorylated at the C-terminus, and the residue also plays a vital role in the response to salt stress [61]. Since there are multiple predicted phosphorylation sites related to CIPKs, their regularity is difficult to conclude in vivo [25]. In the regulatory domain, the autoinhibitory NAF motif has a conserved 21-amino-acid sequence that includes asparagine-alanine-phenylalanine, and it specifically interacts with CBLs to relieve the autoinhibition of the catalytic kinase domain through the coordinated release of the activation loop [57,60,62]. CIPKs have a protein phosphatase interaction (PPI) domain right after the NAF motif, which prompts CIPKs to combine with 2C-type protein phosphatase (PP2C) [63].

Based on amino acid sequence similarity and the number of introns, CIPKs can be divided into intron-rich and intron-poor groups (Figure 2D). Specifically, the intron-rich group, including AtCIPK1, AtCIPK3, AtCIPK8, OsCIPK1, and OsCIPK31 usually contains several introns. Inversely, the intron-poor group contains zero introns or one intron [39,41,64]. Furthermore, analysis of gene duplication indicated tandem duplications only exist in the intron-poor group [65]. Previous studies indicate that the rate of intron gains is lower than the rate of intron losses, and intron number has decreased during recent eukaryote evolution [66]. Therefore, the intron-rich group may comprise the ancestral CIPKs. Moreover, the intron-rich members undergo alternative splicing, which can generate a diversity of transcripts and proteins owing to the occurrence of premature stop codons [67]. In *Arabidopsis*, the splice variants of AtCIPK3 exhibit different expression patterns and downstream partners, which may influence the regulation of Ca^2+^ and ABA signaling [68]. Alternative splicing is a crucial mechanism affecting the CBL–CIPK network. Hence, these findings demonstrate that the structural features of these proteins are the groundwork for further functional characterization of the CBL–CIPK complex, though the structure still needs to be carefully examined and verified in different plants.

**Table 1:** The CBL and CIPK families in plant species.

Species	No. of CBLs	No.of CIPKs	Reference
*Arabidopsis thaliana*	10	26	[38,39]
Canola (*Brassica napus*)	7	23	[29]
Cassava *(Manihot esculenta)*	8	26	[43,44]
Fern (*Selaginella moellendorffii*)	4	5	[13]
Grape (*Vitis vinifera*)	8	20	[37]
Moss (*Physcomitrella patens*)	4	7	[13]
Populus (*Populus trichocarpa*)	10	27	[38,42]
Pepper (*Capsicum annuum*)	9	26	[34]
Rice (*Oryza sativa*)	10	34	[29,39,40,64]
Tea (*Camellia sinensis*)	9	18	[36]
Wheat (*Triticum aestivum)*	24	79	[35,41]

## 3. Mechanisms of the CBL–CIPK Module

As mentioned above, the EF-hand motif in CBLs detects elevated Ca^2+^ levels, and CBLs, in turn, activate the CIPK in response to stimuli. Along with the structural analysis of the CBL–CIPK complex, many studies have suggested that Ca^2+^ plays a crucial role in the mediation of the CBL–CIPK module based on crystal structure analysis [26,69,70]. Experiments with the AtCBL4-AtCIPK24 complex show that Ca^2+^ plays a vital function in this molecular mechanism [70]. However, some results imply that Ca^2+^ is not necessary for the interaction of AtCBLs and AtCIPKs. The NAF motif can bind to AtCBL4 in the presence or absence of Ca^2+^ in vitro [60]. Moreover, the calcium coordination pattern of AtCBL2-AtCIPK14 differs from that of the AtCBL4-AtCIPK24 complex. An EF-hand loss-of-function mutant of AtCBL2 had similar binding affinity compared to normal AtCBL2 [71]. Notably, all these experiments were performed in vitro. Meanwhile, the ability of EF-hand domains to capture Ca^2+^ is influenced by the similar ion Mg^2+^ and the target protein [46]. Therefore, the relationship between Ca^2+^ and sensor proteins still merits further exploration in vivo.

CBLs interact with and activate CIPKs to form complexes, and CIPKs phosphorylate CBLs in the meantime. The phosphorylated CBLs have enhanced functions [69]. In *Arabidopsis*, AtCBL1 is phosphorylated by AtCIPK23 in turn and enhances the activity of AtCIPK23. Phosphorylation of AtCBL1 is important to activate the downstream target AKT1 (*Arabidopsis* K^+^ transporter 1) by using the two-electrode voltage-clamp in *Xenopus* oocytes, a technique that is used to investigate ion channels or transporters [49,72]. In vitro phosphorylation of AtCBL10 (rather than AtCBL4, the other partner of AtCIPK24) is enhanced by its interaction with AtCIPK24 without Ca^2+^. Additionally, Ser^237^ in AtCBL10 is a vital phosphorylation site. When Ser^237^ is mutated to Ala or Asp, the phosphorylation and function of AtCBL10 are changed. Thus, AtCBL10S237A cannot be phosphorylated by AtCIPK24, and AtCBL10S237D can rescue *Atcbl10* salt sensitivity, which demonstrates that phosphorylation has important effects on salt tolerance [73].

Furthermore, AtCBL10 directly interacts with AKT1 and negatively modulates AKT1 activity to influence K^+^ homeostasis, indicating that the target proteins of CBLs are not restricted to CIPKs [74]. In addition to CIPKs, some CBLs in the second group (i.e., AtCBL2, AtCBL3, and AtCBL6) can interact with 5′-methylthioadenosine nucleosidases (MTANs), demonstrating the diversity of targets and processes among CBLs [75].

Similarly, CIPKs interact not only with CBLs, but also with PP2C through the PPI domain. AtCIPK24 associates with the PP2C ABI2 (ABA insensitive 2) to regulate resistance to salt and ABA [63]. AtCIPK9 interacts with AP2C1 (AKT1-interacting PP2C 1) under potassium-deficient conditions in *Arabidopsis* [76]. The PPI motif negatively influences the function of the NAF motif [70]. Apart from PP2C, CIPKs can interact with other proteins. AtCIPK14 phosphorylates the ubiquitin ligase ATL31 (involved in primary nutrient responses) in response to carbon to nitrogen nutrient conditions [77]. An SnRK2s member, SRK2D, interacts with AtCIPK26 and other clade members (AtCIPK3, AtCIPK9 and AtCIPK23) to maintain cellular Mg^2+^ homeostasis [78]. This complicated interaction is also found in other species. Apple (*Malus domestica*) MdCIPK22 can phosphorylate MdAREB2 (ABA-responsive element binding factor) to regulate ABA sensitivity [79]. MdCIPK13 mediates the phosphorylation of MdSUT2.2 (sucrose transporter) protein at Ser^254^ to enhance salt-induced sugar accumulation and salt tolerance [80]. In brief, the complicated structures of CBLs and CIPKs show their wealth of possible interactions. Remarkably, phosphorylation is the main mechanism coordinating the effects of CBL–CIPK on downstream proteins. Phosphorylation sites have been gradually identified in the CBL–CIPK pathway [25]. (We describe the detailed consequences of these findings in the section titled Functions of the CBL–CIPK Pathway.)

## 4. Subcellular Localization of CBLs, CIPKs, and Their Complexes

One CBL can interact with one or multiple CIPKs to form the diverse signaling cascades that lead to the complex CBL–CIPK network. Defining the subcellular localization of CBL and CIPK is imperative to elucidate the functions of CBLs and CIPKs and the mechanisms linking CBLs and CIPKs.

The subcellular localizations of *Arabidopsis* CBLs correspond to their structures. Notably, the groupings of CBLs by structure do not completely match their subcellular localizations. *N*-myristoylation and *S*-acylation modify the protein membrane and regulate the function of proteins [81]. The first group members, namely AtCBL1, AtCBL4, AtCBL5, and AtCBL9, can be localized to the plasma membrane by *N*-myristoylation, which can promote the targeting of proteins to the membrane [54,81]. Additionally, AtCBL4 and AtCBL5 also target the cytoplasm and nucleus. The second group, composed of AtCBL2, AtCBL3, and AtCBL6, which harbor the extended N-terminal domain, is localized to the tonoplast. Specially, AtCBL7 and AtCBL8, members of the second and first groups, respectively, are localized to the cytoplasm and the nucleus [54]. As a member of the third group, AtCBL10 has been reported to be localized to the plasma membrane or tonoplast [53,54,74,82]. The alternative splicing of *AtCBL10* is generated by splicing of the 8th intron, which leads to the different localization patterns [51]. Similar studies have not been reported in other species. In rice (*Oryza sativa*), wheat (*Triticum aestivum*), and poplar (*Populus trichocarpa*), OsCBL4, OsCBL8, and TaCBL1 are plasma membrane-localized proteins, while OsCBL2, OsCBL3, PtCBL10A, and PtCBL10B are localized to the tonoplast [83,84,85,86]. The localizations of most CBLs occur regularly, but some CBLs exhibit specificity that suggests localization but needs to be demonstrated in vivo. Similarly, the function also needs to be clarified in different plant species.

Compared with CBLs, CIPKs are usually localized throughout the cytoplasmic and nuclear compartments without recognizable localization signals [13]. In wheat and pepper (*Capsicum annuum*), TaCIPK14, TaCIPK23, and CaCIPK1 are localized throughout the cell, and CaCIPK5 and CaCIPK20 are only found in the plasma membrane [34,84,87]. In most cases, the subcellular localizations of CBL–CIPK have been demonstrated by bimolecular fluorescence complementation, revealing that CBLs target CIPKs at the plasma membrane or tonoplast to fulfill their functions. In *Arabidopsis*, AtCIPK1 is not only recruited to the plasma membrane by AtCBL1 and AtCBL9, but is also localized to the tonoplast by AtCBL2 [52,88]. Meanwhile, among the other CIPKs, AtCIPK23 is also recruited to the plasma membrane by AtCBL1 and AtCBL9 [89]. In general, CBLs determine the localization of CIPKs in different areas to influence the various functions of the CBL–CIPK complex. However, the localization of the AtCBL10-AtCIPK24 complex has shown contrasting results. Kim et al. reported that AtCBL10 recruits AtCIPK24 to the tonoplast to regulate vacuolar homeostasis of Na^+^. Lin et al. indicated that SOS1, the Na^+^/H^+^ exchange at the plasma membrane, is the target of the AtCBL10-AtCIPK24 complex. The diversified localizations may be activated under different conditions, while the AtCBL10-AtCIPK24 complex requires more investigation. According to the functions of these complexes in plants, CIPKs can interact with other proteins beyond CBLs in their involvement in stress responses, which implies there are multiple localizations of CIPKs.

## 5. Functions of the CBL–CIPK Pathway

**Figure 3:**
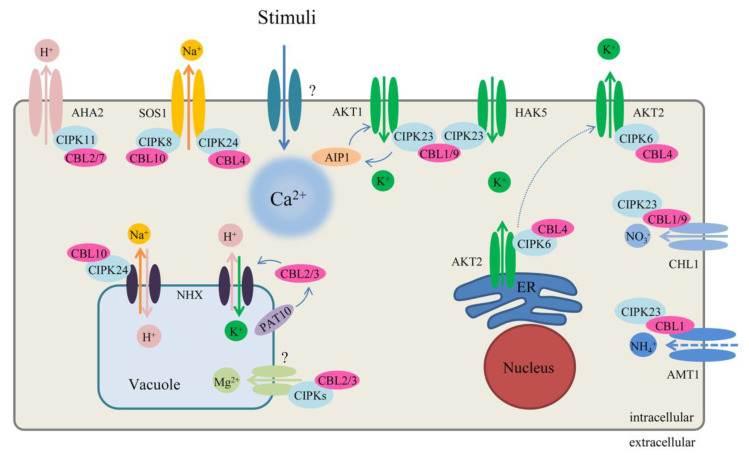
Regulation of the CBL–CIPK network in ion homeostasis in *Arabidopsis*. The solid lines of exchanger at membrane represent the ion direction and interaction, and the dotted line represents negative regulation and moving. See main text for further details. AKT1: *Arabidopsis* K^+^ transporter 1; AKT2: *Arabidopsis* K^+^ transporter 2; AHA2: *Arabidopsis* H^+^ ATPase 2; AMT1: Ammonium transporters 1; AIP1: AKT1-interacting PP2C 1; CHL1: also named NRT1.1, nitrate transporter 1.1; HAK5 high-affinity K^+^ transporter 5; NHX: Na^+^ (K^+^)/H^+^ antiporters; PAT10: Protein S-Acyl transferase 10; SOS1: Salt Overly Sensitive 1, Na^+^/H^+^ exchanger.

**Figure 4:**
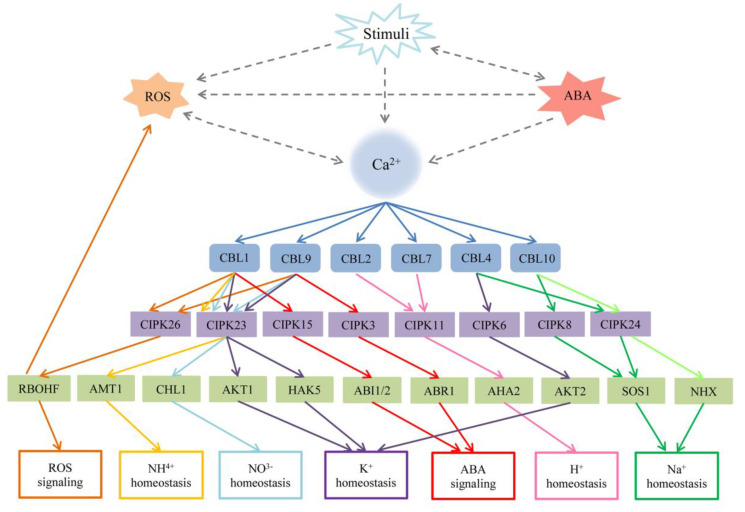
The relationship among Ca^2+^, the CBL–CIPK network, reactive oxygen species (ROS) signaling, abscisic acid (ABA) signaling, and other stresses in *Arabidopsis*. Stimuli usually increase the concentration of Ca^2+^, the production of ROS and ABA in the cytoplasm. Ca^2+^ and ROS promote each other, and the accumulation of ABA will influence the concentration of Ca^2+^ and K^+^. The dotted lines represent indirect connections. The solid lines represent different signaling. AtCBL1 or AtCBL9 interacts with multiple CIPKs to mediate different target proteins in the regulation of many progresses including K^+^, NO_3_^-^, NH_4_^+^ homeostasis, ROS, and ABA signaling. Other CBL–CIPK complexes regulate their related target to involve in H^+^ and Na^+^ homeostasis. ABI1/2: ABA insensitive 1/2; ABR1: Abscisic acid repressor 1; AMT1: Ammonium transporter 1; AKT1: *Arabidopsis* K^+^ transporter 1; AKT2: *Arabidopsis* K^+^ transporter 2; AHA2: *Arabidopsis* H^+^ ATPase 2; HAK5 high-affinity K^+^ transporter 5; NHX: Na^+^ (K^+^)/H^+^ antiporters; RBOHF: respiratory burst oxidase homologs F; SOS1: Salt Overly Sensitive 1, Na^+^/H^+^ exchanger.

CBL–CIPK signaling has been reported to participate in numerous processes associated with responses to abiotic stress (including environmental stress, nutrient deficiency, and phytohormones), biotic stress, ROS signaling, and plant growth and development, which suggests the essential functions of the network throughout the life cycles of plants [2,13,28]. We summarize the most recent investigation of the CBL–CIPK signaling (Figure 3 and Figure 4). Representative results are summarized in Table 2.

### 5.1. CBL–CIPK Pathways in Responses to Abiotic Stress

#### 5.1.1. Environmental Stress

Several environmental factors, such as salt, drought, extreme temperatures, pH stress, and ion toxicity, affect agricultural productivity. Exploring the mechanisms that respond to and enhance resistance to environmental stress is vital to achieving quality and quantity in agricultural production [113]. Drought stress is caused by hyperosmotic stress, and salt stress affects cells through both hyperosmotic stress and ion toxicity. Meanwhile, both salt and drought stresses induce the accumulation of the phytohormone ABA. Therefore, the signals involved in stress sensing and stress responses are complex [113,114]. To date, the response of CBL–CIPK networks to several abiotic stresses have been extensively investigated.

The first study on the CBL–CIPK complex examined the salt overly sensitive (SOS) pathway in *Arabidopsis* and its involvement in salt tolerance. The membrane-localized protein AtCBL4 (SOS3) binds Ca^2+^ via the EF-hand domain and interacts with AtCIPK24 (SOS2) to regulate the plasma membrane-localized Na^+^/H^+^ antiporter (SOS1) [90,115,116]. The *N*-myristoylation of AtCBL4 is also required for regulating ion homeostasis and salt tolerance in plants [117]. SOS1 is phosphorylated at Ser^1138^ and activated by the AtCBL4-AtCIPK24 complex along the plasma membrane, thus increasing salt tolerance [90,118,119]. Overexpression of *SOS1* decreases Na^+^ under high salt conditions, and enhances tolerance to salt stress, suggesting that limiting Na^+^ accumulation in plant cells is important for salt tolerance [120]. The analysis of the gene expression profile in *Atcbl4* and *Atcipk24* mutants indicates that the roles of AtCBL4 and AtCIPK24 in salt stress response are not completely uniform [121]. Independent of AtCBL4, AtCIPK24 interacts with the vacuolar Ca^2+^/H^+^ antiporter CAX1 to impact salt sensitivity [122].

Besides AtCBL4, AtCBL10 (also called SCaBP8, SOS3-like calcium-binding protein) acts as a crucial regulator of salt tolerance. The *Atcbl10* mutant exhibits hypersensitivity to high salt conditions resulting from ionic toxicity. Compared with AtCBL4, AtCBL10 is expressed particularly in shoot tissues rather than root tissues. AtCBL10 also interacts with AtCIPK24 and activates an unknown Na^+^ transporter in the vacuole [53]. The diverse target proteins result in different localization patterns. Specifically, AtCIPK24 phosphorylates AtCBL10 at Ser^237^ under salt stress [53,73,123]. Additionally, AtCIPK8 is homologous to AtCIPK24, which also interacts with AtCBL10 and activates SOS1 to regulate salt tolerance [91]. However, the relationship between AtCIPK8 and AtCIPK24 under high salt conditions is unclear, and the potential functional redundancy of the SOS pathway remains to be examined. In brief, the SOS pathway is a vital regulatory pathway that controls Na^+^/H^+^ homeostasis and salt tolerance in plants (Figure 3). Two 14-3-3 proteins (general regulatory factors with highly conserved domains) have been found to interact with AtCIPK24 and repress its kinase activity. The interactions between AtCIPK24 and 14-3-3 proteins are reduced under salt stress. The Ser^294^ residue of AtCIPK24 is an important phosphorylation site. Mutation of Ser^294^ influences AtCIPK24 activity in the presence of 14-3-3 proteins [124].

*AtCBL1* is highly inducible by multiple stresses, including drought, salt, cold, wounding, and ABA. Overexpression of *AtCBL1* enhances resistance to salt and drought, while reducing resistance to freezing. In contrast, tolerance to freezing is increased in mutant plants, but resistance to salt and drought is reduced, suggesting that AtCBL1 plays diverse and even opposing functions under different stresses [32]. The earliest studies on AtCBL1 indicated that it was a multifunctional and important protein. Subsequently, numerous studies have confirmed this hypothesis [97,125,126,127]. We introduce them in the corresponding section. Another calcineurin B-like protein, AtCBL5, may positively regulate the response to salt and drought. In particular, *AtCBL5* is not induced by salt, drought, or low temperature. *AtCBL5*-overexpression plants have enhanced tolerance to salt and drought [128]. However, few studies have been reported on AtCBL5 and its target protein, which need more attention.

As the interaction partner of CBLs, CIPKs also participate in salt and drought stress responses. AtCIPK6 mediates an endoplasmic reticulum (ER)-localized calcium-binding peptide (CBP) to regulate resistance to salt and drought. The expression of *AtCIPK6* is induced in CBP-overexpressing plants, and the *Atcipk6* mutant plants reduce the improved tolerance of CBP-overexpressing plants [129]. Additionally, AtCIPK21 mediates the response to salt and osmotic stress via its interaction with AtCBL2 and AtCBL3 in the tonoplast [92].

Apart from experiments on *Arabidopsis*, CBLs and CIPKs have been found to respond to salt and drought stresses in other species. In rice, *OsCIPK23* RNAi transgenic plants show a hypersensitive response to drought stress [130]. Overexpression of maize *ZmCIPK8* in tobacco improves drought tolerance and regulates the expression of some stress-related genes [131]. In apple, overexpression of *MdSUT2.2*, a sucrose transporter gene, increases both sucrose accumulation and salt resistance. MdCIPK13 phosphorylates MdSUT2.2 at Ser^254^ and mediates its phosphorylation to improve salt resistance [80]. MdSOS2L1 (MdCIPK24-LIKE1) interacts with several MdCBLs (MdCBL1, -4 and -10). Overexpression of *MdSOS2L1* increases antioxidant metabolites to resist salt stress in both apple and tomato [112]. Additionally, *HbCIPK2* is induced by salt, drought, and ABA treatment in *Hordeum brevisubulatum*. Ectopic expression of *HbCIPK2* enhances salt tolerance in both *Arabidopsis* mutant *sos2-1* and wild-type plants, revealing its role in tolerance to osmotic stress and indicating that *HbCIPK2* positively regulates resistance to salt and osmotic stress [132]. Compared with model plants, it is difficult to perform functional verification in most species because of technical limitations. Therefore, related research on crop demands particularly intensive study.

Chilling and freezing injuries are common in temperate regions. Nevertheless, little is known about the role of CBLs and CIPKs in cold stress responses. In *Arabidopsis*, *AtCIPK3* is responsive to cold, high salt, wounding, drought, and ABA. In *Atcipk3* mutant plants, the expression profiles of several stress-related genes are altered, involving genes related to ABA, cold, and high salt responses, but not drought-induced genes. *AtCIPK7* and *AtCBL1* expression is induced by cold, and AtCIPK7 regulates cold stress responses via its interaction with AtCBL1 [93]. Wheat *OsCIPK03* is induced by cold stress and its overexpression improves cold tolerance [40].

In plants, several factors, including blue light and fungal elicitors, regulate plasma membrane H^+^-ATPases to alter pH [133,134]. Ambient alkaline or acid pH stress in the soil environment impedes the growth and development of plants. *Atcipk11* (also referred to as PKS5, SOS2-like protein kinase 5) mutants can show improved tolerance of high external pH, and AtCIPK11 interacts with AtCBL2/SCaBP1. AtCIPK11 phosphorylates plasma membrane H^+^-ATPases AHA2 (*Arabidopsis* H^+^ ATPase 2) at Ser^931^ and prevents interaction between AHA2 and an activated 14-3-3 protein [94]. In a recent report, an *Atcbl7* mutation enhanced stress tolerance via increasing plasma membrane H^+^-ATPase activity. AtCBL7 interacts with AtCIPK11 to stabilize kinase-ATPase and inhibit the phosphorylation of AHA2 [135].

Metals, such as magnesium (Mg), aluminum (Al), mercury (Hg), cadmium (Cd), and chromium (Cr), are involved in enzymatic activity and regulate reactive oxygen species (ROS), which influences crop yields [136]. As an essential macronutrient for plants, Mg deficiencies cause leaf chlorosis, but excessive Mg is toxic to plant cells [137]. In *Arabidopsis*, some homologous AtCIPKs (3, -9, -23, and -26) function as overlapping components via their interactions with AtCBL2 and AtCBL3 and phosphorylation with SRK2D/E/I proteins to maintain Mg^2+^ homeostasis across the vacuolar membrane. Remarkably, these homologous proteins modulate Mg^2+^ homeostasis in a redundant manner [30,78]. Al is the most widely distributed metal in the environment, but it severely impacts root growth and functions. In *Arabidopsis cbl1* mutants, Al sensitivity is enhanced compared with wild-type plants and is regulated by reduced malate efflux, leading to higher Al accumulation in roots and shoots. The same function is not found for the homologous gene *AtCBL9* [126]. In annual wild barley (*Hordeum spontaneum*) grown on the Tibetan Plateau, multiple *HsCIPKs* (*HsCIPK-2*, *-5*, *-17*, *-28*, *-29*, and *-30*) are regulated by abiotic stresses, including multiple heavy metals, and overexpression of each *HsCIPK* in rice enhances root tolerance to heavy metal toxicity, salt stress, and drought stress [138]. The relationship among *HsCIPKs* in the regulation of heavy metal responses should be examined in depth. Apart from known SOS pathways, the mechanisms of other abiotic stresses are still unclear, requiring further research.

#### 5.1.2. Nutrient Deficiency in Plants

Plant roots absorb water and nutrients from the soil enabling them to grow and develop. Soil nutrients include macronutrients, such as nitrogen, potassium, phosphorous, calcium, and magnesium, and micronutrients, such as iron, boron, and manganese [2,139,140].

Nitrate (NO_3_^−^) is the primary nitrogen source for plants growing in soil, and it also acts as a signaling molecule [140,141]. Plants absorb nitrogen from soil by means of two systems, namely high- and low-affinity systems [142]. In *Arabidopsis*, *AtCIPK8* is upregulated by nitrate, and experiments with knockout mutants and complementation experiments demonstrate that AtCIPK8 is involved in long-term nitrate-modulated primary root growth targeting by NRT1.1 (Nitrate transporter 1.1, also named CHL1), which has a vital role in nitrate transport [143]. CHL1 functions as a nitrate sensor and is phosphorylated by AtCIPK23 at Thr^101^ dependent upon the activation by AtCBL1 and AtCBL9 to regulate the response to low nitrate conditions [96]. Meanwhile, loss-of-function mutations in *Atcipk23* increase NH_4_^+^ concentrations in roots under high external ammonium conditions. The high-affinity AMT1 (Ammonium transporter 1) is inactivated by phosphorylation at Thr^460^ to avoid the toxic accumulation of high ammonium concentrations. AtCIPK23 inhibits AMT1 activity by interacting with AtCBL1 without AtCBL9 [97,144].

Potassium (K^+^) is a crucial macronutrient in the soil, and it plays an important function in cell metabolism and growth. The functions of potassium are related to electrical charge balancing, photosynthesis, stomatal movement, long-distance transport, enzymes, and protein synthesis [145,146,147]. Plant roots acquire K^+^ through high- and low-affinity transport systems [148]. Low-K conditions can cause ROS accumulation, followed by activation of Ca^2+^. Then, AtCIPK23, which is encoded by the *LKS1* (low-K^+^-sensitive 1) gene, directly phosphorylates AKT1 through activation by the homologous proteins AtCBL1 and AtCBL9 under low-K^+^ conditions [89,98]. The predicted phosphorylation site of AKT1 is Ser^744^, though this has not been empirically confirmed [25]. Similar regulatory mechanisms exist in rice root tissues; the OsAKT1 channel mediates K^+^ uptake dependent on the OsCBL1-OsCIPK23 complex [108]. *AtKC1* (K^+^ rectifying channel 1) improves the tolerance of low K^+^ conditions and increases K^+^ accumulation. AtKC1 negatively regulates K^+^ modulated mediation by AKT1 with AtCIPK23 synergistically and controls the root-to-shoot ratio under low K^+^ levels [95,149]. In addition, HAK5 (High-affinity K^+^ transporter 5), a major system mediating K^+^ uptake, is also phosphorylated by AtCIPK23 to control high-affinity K^+^ uptake in root tissues [99]. AtCIPK23 has been observed to activate its target proteins AKT1 and HAK5. AKT1 has been speculated to be active under mild K^+^ deprivation, while HAK5 is hypothesized to be fully active under severe K^+^ deprivation [99]. Furthermore, a loss-of-function mutation of *Atcipk23* enhances sensitivity to iron deficiency and changes the concentration of mineral elements. Under low iron, elevated levels of Ca^2+^ bind AtCBL1/AtCBL9 to activate AtCIPK23 [150]. Based on research on nitrate transport, the CBL1/CBL9-CIPK23 complexes appear to act as “nutritional sensors” to regulate potassium, nitrate, and iron processes in plant growth and development [150,151]. *AtCIPK23* contains several introns and can produce activated isoforms, which may underlie multiple functions of the proteins it encodes [39]. The mechanisms by which AtCIPK23 and its different target proteins respond to various stresses needs more study in vivo. In addition to AtCBL1 and AtCBL9, AtCBL10 also regulates AKT1 activity. Overexpression of *AtCBL10* induces a low K^+^-sensitive phenotype as *akt1* mutant seedlings. AtCBL10 negatively alters the activity of AKT1 on the plasma membrane through interactions without any CIPKs, which affects K^+^ homeostasis [74]. Since CIPKs contain a PPI motif, PP2CA (PP2C member) interacts with the AtCIPK6 kinase domain to inactivate AKT1. Additionally, several AtCBLs negatively regulate the activity of PP2CA to reduce interactions between PP2CA and AtCIPK6, thus enhancing the activation of AKT1 [152]. These results indicate that AtCBLs can indirectly regulate CIPKs through PP2C. As a PP2C protein family member, AIP1 interacts with AtCIPK23, and both interact with AKT1 to modify its activity by phosphorylation and dephosphorylation [153]. A recent study has reported that the 2C-type phosphatase protein AP2C1 interacts with AtCIPK9 in the cytoplasm and dephosphorylates the autophosphorylated AtCIPK9 to regulate K^+^-deficiency responses [76]. Moreover, another K^+^ channel, AKT2, has been found to regulate the CBL–CIPK complex. AKT2 has a distinct function compared with other K^+^ channel subunits, suggested by the weak inward-rectifying activity observed in *Xenopus* oocytes [154]. AtCBL4 interacts with AtCIPK6 and modulates the targeting of AKT2 from the ER to the plasma membrane via K^+^ channels [100] (Figure 3). Although most studies focusing on K^+^ homeostasis center on the CBL1/CBL9-CIPK23 complex, the complicated relationships among CBL, CIPK, and other downstream proteins still remain to be explored.

#### 5.1.3. Plant Hormone ABA

The phytohormone ABA serves as an endogenous messenger in responses to drought, high salinity, and cold, and it participates in diverse growth and physiology pathways [155]. Many studies have shown that osmotic stress is regulated by ABA-dependent and ABA-independent pathways [156]. In *Arabidopsis*, *AtCIPK3* is upregulated by cold, salt, and ABA treatment. *Atcipk3* mutant plants show different expression patterns of stress- and ABA-induced genes, suggesting *AtCIPK3* acts as a crosstalk “node” between the ABA and stress pathways. Meanwhile, AtCBL9 also modulates the ABA signaling and biosynthesis pathways and interacts with AtCIPK3 in response to ABA signaling during seed germination [33,157,158]. AtCIPK3 has been shown to interact with ABR1 (Abscisic acid repressor 1) to regulate ABA response, leading to the conclusion that the CBL9-CIPK3-ABR1 pathway regulates ABA-dependent physiological processes and seed germination [102]. Based on its abundance of introns, AtCIPK3 was selected for examination of alternative splicing. Five splice variants of AtCIPK3 have shown different expression patterns and downstream proteins, which provides new insights into mechanisms related to Ca^2+^ [68].

The vacuolar membrane-localized AtCBL2 with *S*-acylation plays an essential role in ABA responses. PAT10 (Protein *S*-Acyl transferase 10) regulates several AtCBLs (AtCBL2, -3, and -6) at the tonoplast and plays a critical role in the development and salt tolerance in *Arabidopsis* [159,160]. ABA also increases the concentration of Ca^2+^ and K^+^ in the cytoplasm by stomatal movement [31]. Recent research has demonstrated that the PAT10-AtCBL2/3-AtCIPK9/17 system negatively regulates ABA signaling during stomatal movement. Meanwhile, Na^+^ (K^+^)/H^+^ antiporters (NHXs) are regulated by PAT10 and AtCBL2/3 to control K^+^ homeostasis [30,31]. The complex of AtCBL1/CaBP5-AtCIPK15/PKS3 is also involved in the ABA signaling pathway, and AtCIPK15 associates with the 2C-type protein phosphatase ABI1 and ABI2 [103]. Additionally, CIPK11/PKS5 phosphorylates ABI5 at Ser^42^ to regulate ABI5 activity and positively function in plant ABA signaling [104].

The guard cell anion channel, S-type anion channels SLAC1 (Slow Vacuolar Anion Channel 1), and SLAH3 (SLAC1 homolog 3) are key components of calcium-independent ABA signaling to regulate the opening and closing of stomata [161]. AtCBL1/AtCBL9-AtCIPK23 complexes trigger SLAC1 and SLAH3 in *Xenopus* oocytes. Thr^513^ is an important phosphorylation site of SLAC1, whereas the activation of SLAC1 can be inhibited by the phosphatase ABI1 (ABA insensitive 1) [101]. SLAC1 is also activated by the AtCBL5-AtCIPK11 complex along the plasma membrane [81]. Notably, ABA accumulation leads to the inactivation of ABI2 by the RCAR/PYL/PYR system, following the enhanced phosphorylation of CHL1 by AtCBL1-AtCIPK23, which regulates nitrate and potassium uptake [151]. Thus, the CBL–CIPK network is involved in ABA signaling as well as nitrate and potassium homeostasis simultaneously, which indicates the core role of the CBL–CIPK network in plant signaling.

Apple MdCIPK22 interacts with and phosphorylates MdAREB2 to regulate the ABA signaling pathway [79]. In wheat, the transcriptional levels of *TaCIPK14* and *TaCIPK27* are upregulated under ABA treatment [87,162]. Overexpression of *TaCIPK27* improves drought tolerance but sensitizes plants to exogenous ABA treatment in *Arabidopsis*, suggesting that TaCIPK27 positively regulates drought stress in an ABA-dependent pathway [162]. Wheat *TaCIPK23* is a positive regulator in drought stress and ABA signaling [84]. The diversified functions of the CBL–CIPK complexes in plant signaling indicate that the network deserves further study.

### 5.2. The CBL–CIPK Network in Biotic Stress

Plant growth and development are affected by various biotic stresses, such as pathogenic microbes, parasitic plants, and herbivores [163,164]. Pathogens usually cause significant damage to plant yields. The innate immunity of plants is divided into two forms: pattern-triggered immunity (PTI) and effector-triggered immunity (ETI). PTI results from PRR-mediated recognition of microbe-associated molecular patterns (MAMPs) and damage-associated molecular patterns (DAMPs), which promote basal immunity, whereas ETI is based on interactions with pathogen effectors and causes a hypersensitive response to limit pathogen spread [165,166]. Plants generate ROS as byproducts of various aerobic metabolic processes in mitochondria, chloroplasts, and peroxisomes. Meanwhile, ROS function as signaling molecules in response to pathogen infection and programmed cell death under biotic stress conditions and regulates Ca^2+^ release. NADPH oxidases, also referred to as respiratory burst oxidase homologs (RBOH), are closely linked to ROS production [167,168,169].

*Arabidopsis* AtCIPK6 negatively regulates ROS production in ETI and PTI. *Atcipk6* loss-of-function mutant plants possess more resistance to *Pseudomonas syringae*, but overexpression lines are more susceptible [170]. In rice, *OsCIPK14* and the duplicated gene *OsCIPK15* are rapidly induced by MAMPs, and their proteins interact with OsCBL4. RNAi lines of *OsCIPK14* and *OsCIPK15* exhibited reduced sensitivity to TvX/EIX (*Trichoderma viride*/ethylene-inducing xylanase), whereas this phenotype was enhanced in *OsCIPK15*-overexpression lines, suggesting that the two genes are crucial in MAMP defense signaling pathways [107]. In the vital food crop cassava (*Manihot esculenta*), *MeCIPK23* is induced by *Xanthomonas axonopodis* pv. *manihotis* (*Xam*) and interacts with MeCBL1 and MeCBL9. Overexpression of each gene induces resistance to *Xam*. In contrast, silenced plants are sensitive to *Xam*, indicating that MeCBL1/MeCBL9-MeCIPK23 complexes play important functions in the defense response [111].

AtCIPK26 is recruited by AtCBL1 and AtCBL9 to the plasma membrane, and phosphorylates the NADPH oxidase AtRBOHF. Coexpression of either AtCBL1 or AtCBL9 with AtCIPK26 increases ROS accumulation by RBOHF in heterologous HEK293T cells, suggesting that AtCBL1/AtCBL9-AtCIPK26 complexes phosphorylate RBOHF to regulate ROS signaling [105,171,172]. AtRBOHF is regulated by AtCIPK11, AtCIPK26, and the ABA-dependent kinase AtOST1 (open stomata 1). In HEK293T cells, AtCIPK11 and AtCIPK26 constitute alternative pathways for AtRBOHF activation. AtRBOHF contains three phosphorylation sites (Ser^13^, Ser ^130^, and Ser^132^) at the N-terminus. The activation of AtRBOHF also enhanced the combined function of AtOST1 and AtCIPK26. Additionally, the ABA insensitive phosphatase ABI1 can dephosphorylate AtRBOHF and counteract kinase-mediated activation [105]. These results indicate that crosstalk among the CBL–CIPK network, ROS, and ABA signaling pathways merit further examination.

In wheat, *TaCBL4* transcript levels increase rapidly after infection by *Puccinia striiformis* f. sp. *tritici* (*Pst*) and interact with TaCIPK5. Resistance to *Pst* is decreased in *TaCBL4*-silenced plants along with ROS reduction. The same phenotype is observed in *TaCIPK5*-silenced plants and cosilenced plants. Together, the TaCBL4-TaCIPK5 complex positively regulates tolerance to stripe rust fungus dependent on ROS signals [35]. Overexpression of wheat *TaCIPK10* improves tolerance to *Pst* by hypersensitive cell death and ROS accumulation. TaNH2, a homolog of *Arabidopsis* NPR3/4, which is involved in salicylic acid (SA) signaling, combines with TaCIPK10 to participate in the defense response through interaction and phosphorylation [106]. In tomato (*Solanum lycopersicum*), SlCBL10 interacts with SlCIPK6 and regulates the kinase activity of SlCIPK6. Overexpression of *SlCIPK6* in *Nicotiana benthamiana* induces ROS production via the regulation of NbRBOHB and the kinase activity of SlCIPK6. Meanwhile, the SlCBL10-SlCIPK6 complex interacts with the downstream protein RBOHB and contributes to increases in ROS involved in plant immunity [110]. Pepper *CaCIPK1* is regulated by diversified stresses, such as *P. capsici* and SA. Silencing of *CaCIPK1* enhances the tolerance of *P. capsici*, and transient expression induces ROS (e.g., H_2_O_2_) generation, suggesting that *CaCIPK1* modulates *P. capsici* tolerance by ROS and SA signaling [34]. Notably, ROS production under biotic stress can promote Ca^2+^ release in neighboring cells to activate Ca^2+^ channels in turn, suggesting the crucial functions of the CBL–CIPK network in ROS-mediated signaling pathways (Figure 4).

### 5.3. The Role of CBL–CIPK Network in Plant Development

Plant growth and development are composed of many processes, spanning the stages of seed germination, seedling growth, flowering, fruiting, senescence, and death. In flowering plants, floral development is integral to plant reproduction and is sensitive to environmental stresses such as high temperature, drought, and cold [173]. The stigma captures pollen and mediates the pollen tube to deliver sperm toward the ovule [174,175]. Studies of specific CBLs and CIPKs have presented functional insights into developmental processes.

*Arabidopsis* AtCBL1 and AtCBL9 are involved in pollen germination and growth. Overexpression of *AtCBL1* or *AtCBL9* impairs pollen morphology and even germination under high external K^+^ concentrations. Although AtCBL1 and AtCBL9 are involved in the regulation of K^+^ homeostasis, ABA signaling, and NO_3_^-^ homeostasis, the relationships among the processes are still unclear and worthy of further research [96,98,125,157]. *AtCIPK19* is detected in pollen grains and pollen tubes specifically at much higher levels than in other tissues. *AtCIPK19* overexpression, mutant, and complementation lines exhibit differences in tube growth and polarity, indicating that AtCIPK19 is required for these processes [175]. The loss-of-function *Atcbl10* mutant has reduced anther dehiscence, shortened stamen filaments, aborted pollen development, and a lack of pollen tube germination. Hence, *AtCBL10* is important for reproductive development under salt stress [176]. Wheat *OsCIPK23* is upregulated by multiple stresses and has a unique function in pollination and drought tolerance [130].

Flowering time gene GIGANTEA (GI) has a major role in photoperiodicity and circadian control. AtCIPK24 is captured by GI instead of SOS1 under normal conditions, but the complex is degraded in the presence of salt, which provides a unique mechanism between plant development and environmental stress in *Arabidopsis* [177]. Tomato *SlCIPK2* is specifically detected in floral organs and interacts with several SlCBLs and some stress-responsive transcription factors, suggesting that *SlCIPK2* is involved in stamen development and stress tolerance via calcium signaling [173].

Root morphology and distribution are essential to acquire water and nutrients from soil to sustain plant growth and productivity [178]. The *Arabidopsis Atcipk6* mutant exhibits altered auxin transport among plant organs as well as developmental defects and salt sensitivity. Overexpression of the homologous gene (*CaCIPK6*) from chickpea (*Cicer arietinum*) influences auxin transport basipetally, revealing novel functions in auxin transport and root development [179]. Under low-salt conditions, an *Atcbl4* mutant demonstrated the novel role of *AtCBL4* in regulating lateral root development. Mutant plants have reduced auxin content in their cotyledons and lateral root primordia, which may lead to defects in lateral root initiation and cell division activity in lateral root primordial [180]. *AtCIPK25* expression shows the opposite pattern under auxin and cytokinin treatments; mutant plants exhibit a short root phenotype owing to slow root growth rates and regulation of the expression of *PIN1* (auxin efflux carriers), *PIN2*, and *SHY2* (Aux/IAA family gene). Combined with evidence from the *shy2* loss-of-function mutation, *PIN1* and *PIN2* expression in an *Atcipk25* mutant line were recovered along with a normal root phenotype, indicating that *AtCIPK25* is involved in root meristem development through auxin and cytokinin signaling [181].

Sugars modulate most processes in plant development, such as seed germination and seedling development [182,183,184]. *AtCBL1* is regulated by glucose, and mutant lines were more sensitive to glucose and paclobutrazol (a gibberellin biosynthesis inhibitor). In addition, AKINβ1, an important gene in sugar signaling, interacts with AtCBL1, suggesting that AtCBL1 has a novel function in the regulation of glucose and gibberellin signals [127]. Recently, cotton (*Gossypium hirsutum*) GhCBL2 has been shown to recruit GhCIPK6 to the tonoplast and interact with TST2 (tonoplast-localized sugar transporter 2) to regulate sugar homeostasis [109]. Thus, uncovering the downstream components of CBL–CIPK signaling may help to expand in the current understanding of plant growth and development.

## 6. Conclusions and Perspectives

**Figure 5:**
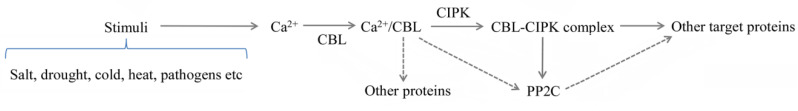
A model of CBL-CIPK pathway during stimuli. CBLs combine with the Ca^2+^ increased by stimuli, and activate the CIPKs in response to relevant stresses. The solid lines represent the classical Ca^2+^/CBL-CIPK signaling. The atypical function of CBLs and the target protein PP2C are shown with dotted lines.

Environmental stresses are the major factors impeding plant growth and development processes, affecting crop productivity worldwide. Calcium signaling has a significant role in many adaptations and developmental processes in plants. In recent years, the CBL–CIPK network has been extensively researched to establish a firm foundation enabling research progress, especially in the model plant *Arabidopsis* and the staple food crops wheat and rice. CBL and CIPK family members have been discovered widely in different plant species, but it remains unclear which members play essential roles in stress responses. The relationships among CBLs, CIPKs, and other target proteins in responses to diverse forms of stress have been gradually clarified (Figure 5). Classical CBL–CIPK signaling consists of Ca^2+^, CBLs, CIPKs, and target proteins. CBLs bind with Ca^2+^ induced by stimuli and interact with CIPKs to activate various downstream target proteins. Furthermore, some atypical functions have been gradually discovered in plants. CBLs interact with other proteins such as AKT1, PAT10, and MTANs in addition to CIPKs [74,75,160]. Moreover, some CBLs are reported to solely regulate stress in calcium signaling pathways, but details about the upstream and downstream components remain to be determined. Notably, CBLs interact with and inhibit the PP2C member that influences interactions with CIPKs [152].

Several CBLs interact with single or multiple CIPKs, such as AtCBL1/9 with AtCIPK23, AtCBL2/3 with AtCIPK9/17, and AtCBL4/10 with AtCIPK24 in *Arabidopsis*. The complicated and various interactions in the responses to different conditions result in crosstalk to parallel or competing pathways involved in plant development. In particular, the AtCBL1/9-AtCIPK23 complex functions in the regulation of iron processes (potassium and nitrate), ABA signaling, and plant growth and development. To fully elucidate the functions of these signaling pathways and understand how the specificity and overlap of functions contribute to their related functions, the regulation mechanisms should be defined. Despite major advances in research on abiotic stress, including salt, drought, and ion stress, the functions of CBLs and CIPKs in biotic stress and plant development require further detailed study.

Based on the well-known regulatory functions of CBLs and CIPKs, there are still many questions to be answered (1) What is the mechanism of binding affinity between Ca2+ signals and CBLs? (2) How do CBLs influence CIPKs and their target proteins? (3) What are the functions of CBLs with other proteins, beyond the classically linked CIPKs? (4) How do the multiple CBL–CIPK networks function under the same stress in vivo? (5) What mechanisms remain undiscovered between the CBL–CIPK network and other signaling pathways? (6) Despite normal membrane system conditions, can CBLs or CIPKs play functions in other localizations? (7) What potential functions are enabled by alternative splicing in CBLs and intron-rich CIPKs? More generally, the calcium-mediated CBL–CIPK network is worthy of exploring for in-depth insights into plant responses to stress signaling via biotechnological approaches, such as biosensors, phosphorylation assay, crystallography, etc.

## Figures and Tables

**Table 2:** Overview of CBL–CIPK modules in plants.

Species	CBL	CIPK	Target Protein	Localization of Complex	Stimulus or Pathway Affected	Reference
*Arabidopsis thaliana*	AtCBL4/SOS3	AtCIPK24/ SOS2	SOS1	Plasma membrane	Salt stress	[90]
	AtCBL10		-	Tonoplast/Plasma membrane	Salt stress	[38,73]
	AtCBL10	AtCIPK8	SOS1	Plasma membrane	Salt stress	[91]
	AtCBL2/-3	AtCIPK21	-	Tonoplast	Salt and osmotic stress	[92]
	AtCBL1	AtCIPK7	-	-	Cold	[93]
	AtCBL7	AtCIPK11	AHA2	Plasma membrane	H^+^ homeostasis	[94]
	AtCBL2/-3	AtCIPK3/-9/ -23/-26	SRK2D/E/I	Tonoplast	Mg^2+^ homeostasis	[30,78]
	-	AtCIPK8	CHL1	-	NO_3_^−^ homeostasis	[95]
	AtCBL1/-9	AtCIPK23	CHL1	Plasma membrane	NO_3_^−^ homeostasis	[96]
	AtCBL1	AtCIPK23	AMT1	Plasma membrane	NH_4_^+^ homeostasis	[97]
	AtCBL1/-9	AtCIPK23	AKT1	Plasma membrane	K^+^ homeostasis	[89,98]
	AtCBL1/-9	AtCIPK23	HAK5	Plasma membrane	K^+^ homeostasis	[99]
	AtCBL10	-	AKT1	Plasma membrane	K^+^ homeostasis	[74]
	AtCBL4	AtCIPK6	AKT2	Plasma membrane	K^+^ homeostasis	[100]
	AtCBL1/-9	AtCIPK23	SLAC1/ SLAH3	-	ABA signaling	[101]
	AtCBL5	AtCIPK11	SLAC1	Plasma membrane	ABA signaling	[81]
	AtCBL2/-3	AtCIPK9/-17	-	Tonoplast	ABA signaling	[31]
	AtCBL9	AtCIPK3	ABR1	-	ABA signaling	[102]
	AtCBL1	AtCIPK15	ABI1/ABI2	-	ABA signaling	[103]
	-	AtCIPK15	ABI5	-	ABA signaling	[104]
	AtCBL1/-9	AtCIPK26	RBOHF	Plasma membrane	ROS signaling	[105]
*Triticum aestivum*	TaCBL4	TaCIPK5	-	-	*Pst*	[35]
	-	TaCIPK10	TaNH2	-	*Pst*	[106]
*Oryza sativa*	OsCBL4	OsCIPK14/-15	-	-	*TvX/EIX*	[107]
	OsCBL1	OsCIPK23	OsAKT1	Plasma membrane	K^+^ homeostasis	[108]
*Gossypium hirsutum*	GhCBL2	GhCIPK6	GhTST2	Tonoplast	Sugar homeostasis	[109]
*Solanum lycopersicum*	SlCBL10	SlCIPK6	RBOHB	Plasma membrane	ROS signaling	[110]
*Manihot esculenta*	MeCBL1/-9	MeCIPK23	-	Plasma membrane	*Xam*	[111]
*Malus domestica*	-	MdCIPK13	MdSUT2.2	-	Salt stress	[80]
	MdCBL1/-4/-10	MdSOS2L1	-	-	Salt stress	[112]
	-	MdCIPK22	MdAREB2	-	ABA signaling	[79]

“–” represents no data available.

## Abbreviations

ABA	Abscisic acid
ABR	Abscisic acid repressor
ABRE	Abscisic acid responsiveness element
ABI	Abscisic acid insensitive
AHA2	*Arabidopsis* H^+^ ATPase 2
AKT	*Arabidopsis* K^+^ transporter
AIP1	AKT1-interacting PP2C 1
AMT1	Ammonium transporter 1
AREB	ABA-responsive element binding factor
CAMs	Calmodulins
CBLs	Calcineurin-B-like proteins
CBP	calcium-binding peptide
CDPKs	Calcium-dependent protein kinase
CMLs	CaM-like proteins
CIPKs	CBL-interacting protein kinases
DAMP	Damage-associated molecular pattern
EF-hand	Elongation factor-hand
ER	Endoplasmic reticulum
ETH	Ethylene
ETI	Effector-triggered immunity
HAK5	High-Affinity K^+^ transporter 5
KC1	K^+^ rectifying Channel 1
LKS1	Low-K^+^-sensitive 1
MAMPs	Microbe-associated molecular patterns
PAT	Protein S-acyl Transferase
PCD	programmed cell death
PP2Cs	Type 2C protein phosphatases
PPI	Protein phosphatase interaction
PTI	Pattern-triggered immunity
RBOH	Respiratory burst oxidase homologs;
ROS	Reactive oxygen species
SA	Salicylic acid
SARE	Salicylic acid responsiveness element
SLAC	Slow vacuolar anion channel
SnRK3	SNF1-related serine/threonine kinases group 3
SOS	Salt overly sensitive
SUT	Sucrose transporter
TST2	Tonoplast-localized sugar transporter 2
